# The effect of multi-ingredient intra- versus extra-cellular buffering supplementation combined with branched-chain amino acids and creatine on exercise-induced ammonia blood concentration and aerobic capacity in taekwondo athletes

**DOI:** 10.1186/s12970-021-00451-3

**Published:** 2021-06-14

**Authors:** Krzysztof Durkalec-Michalski, Krzysztof Kusy, Natalia Główka, Jacek Zieliński

**Affiliations:** 1 Department of Sports Dietetics, Poznan University of Physical Education, Królowej Jadwigi 27/39, 61-871 Poznań, Poland; 2 Department of Human Nutrition and Dietetics, Poznan University of Life Sciences, Wojska Polskiego 31, 60-624 Poznań, Poland; 3 Department of Athletics, Strength and Conditioning, Poznan University of Physical Education, Królowej Jadwigi 27/39, 61-871 Poznań, Poland

**Keywords:** Supplements, Sport, Ergogenic support, Exercise capacity, Combat sports

## Abstract

**Background:**

This study aimed to investigate the effect of multi-ingredient intra- (BA) versus extra- (ALK) cellular buffering factor supplementation, combined with the customary intake of branched-chain amino acids (BCAA) and creatine malate (TCM), on body composition, exercise variables, and biochemical and hematological parameters in 9 elite taekwondo athletes.

**Methods:**

Eight-week randomized double-blind crossover BA (5.0 g·day^−1^ of β-alanine) versus ALK (0.07 g·kg_FFM_^−1^·day^−1^ of sodium bicarbonate) supplementation combined with BCAA (0.2 g·kg_FFM_^−1^·day^−1^) and TCM (0.05 g·kg_FFM_^−1^·day^−1^) during a standard 8-week taekwondo training period was implemented. In the course of the experiment, body composition (dual X-ray absorptiometry), aerobic capacity (ergospirometric measurements during an incremental treadmill test until exhaustion), and exercise blood biomarkers concentrations were measured. Data were analyzed using repeated measures within-between interaction analysis of variance with the inclusion of experimental supplementation order.

**Results:**

The maximum post-exercise blood ammonia concentration decreased in both groups after supplementation (from 80.3 ± 10.6 to 72.4 ± 10.2 µmol∙L^−1^, *p* = 0.013 in BA; from 81.4 ± 8.7 to 74.2 ± 8.9 µmol∙L^−1^, *p* = 0.027 in ALK), indicating reduced exercise-related adenosine triphosphate degradation. However, no differences were found in body composition, aerobic capacity, blood lactate concentration, and hematological parameters after neither BA (combined with BCAA and TCM) nor ALK (combined with BCAA and TCM) supplementation.

**Conclusions:**

In highly trained taekwondo athletes, neither extra- nor intracellular buffering enhancement resulting from BA and ALK supplementation, combined with BCAA and TCM treatment, affects body mass and composition, maximum oxygen uptake, and hematological indices, even though certain advantageous metabolic adaptations can be observed.

**Supplementary Information:**

The online version contains supplementary material available at 10.1186/s12970-021-00451-3.

## Background

Muscle acidosis, contributing to peripheral and central fatigue, is a crucial problem in sports practice. Experiments at the cellular and molecular level showed that decreased pH and elevated inorganic phosphate negatively affect the force-power curve produced by twitching muscle fibers, their maximum shortening velocity, and motility speed [1]. The exercise-induced acid-base imbalance is a substantial challenge for elite athletes [2–4]. These and other fatigue-related phenomena result in a decrease in physical performance in humans. Acid-base disturbances may be diagnosed with the usage of highly sensitive markers, such as blood ammonia concentration. Ammonia is a waste product of the metabolism of nitrogenous compounds and is considered both a central and peripheral factor behind exercise-induced fatigue. It is produced via the breakdown of branched-chain amino acids (BCAA) in skeletal muscle and the deamination of adenosine monophosphate during exercise [5]. In turn, ammonia metabolism is integrated with the function of kidneys. When acid-base homeostasis is maintained, ammonia metabolism involves both renal and extrarenal pathways [6]. Thus, the implementation of different buffering supplements into athletic training should be supported with the simultaneous analysis of markers such as ammonia.

Many supplementation protocols are often isolated from ergogenic aids that athletes customarily implement. Consequently, they do not reflect the features of competitive sport, omitting additive (synergistic or counteractive) effects of combined use of different supplements, repeated use of supplements, or individual responsiveness [7]. Sports practice observations and previous studies on the efficacy of the use of supplements indicate that BCAA, creatine (CR), beta-alanine (βA), and alkaline agents such as sodium bicarbonate (SB) may be considered as ergogenic support in combat sports [8–11]. BCAAs, especially leucine, have been reported to be responsible for protein synthesis stimulation and/or promoting the anti-catabolic effect [12, 13].

CR is commonly used to increase phosphocreatine (PCr) concentration [14], its resynthesis rate [14], and, consequently, enhance exercise capacity [12]. Moreover, CR supplementation supports the training-induced increases in lean body mass [14], strength [14], and performance in short-duration maximal-intensity exercise [14], which is particularly important in speed-power sports [12, 14]. Nowadays, the most clinically effective and extensively studied form of CR, in terms of performance improvements, is creatine monohydrate [12]. Nevertheless, creatine malate (TCM) seems to be more popular among combat sports athletes, based on the belief about lower water retention in comparison to creatine monohydrate. Although this opinion is widespread in the sports community, there is no conclusive scientific evidence to support it.

βA is a non-essential amino acid that serves as an intracellular buffer [15–18] in muscles, counteracting exercise-induced homeostasis disturbances [12]. It can reduce neuromuscular fatigue, improve performance in exercise bouts lasting up to 4 min, increase the possible number of repetitions or training volume, and indirectly affect lean body mass [12]. βA is a rate-limiting precursor in the synthesis of β-Alanyl-L-histidine (carnosine) [19]. The increases in intramuscular carnosine can attenuate acidosis and fatigue [19]. Its main role in muscles is to attenuate exercise-induced reductions in pH [19].

SB is a primary extra-cellular buffer [15, 20–22] of the efflux of H^+^ ions from contracting muscles to the blood that decreases acidification during high-intensity exercise. It also reduces fatigue during and after anaerobic exercise [12, 23]. SB supplementation results in blood alkalosis, leading to increased efflux of H^+^ and lactate from muscles [24]. Metabolic alkalosis, caused by elevated HCO_3_^−^ concentration, upregulates the activity of the glycogenolysis, thus may boost the utilization of muscle glycogen stores during exercise [24, 25]. SB supplementation improves speed, muscle power, and performance in speed-power sports [12, 25].

There is still a need for research on the simultaneous use of different ergogenic supplements (like BCAA and CR) and their combined effect with an additional component influencing the buffering potential on exercise adaptation and performance among competitive professional athletes. To our knowledge, combined supplementation protocols were only used in a scarce number of studies including elite taekwondo athletes [9, 26]. Thus, this study aimed to find out whether multi-ingredient intra- (BA) or multi-ingredient extra- (ALK) cellular buffering treatment in combination with customary BCAA and TCM supplementation has a noticeable effect on body composition, aerobic capacity, respiratory indices, and blood exercise-related and hematological biomarkers in highly trained taekwondo athletes. According to our previous observations [8], we hypothesized that several weeks of combined intra- (BA) cellular buffering agent, BCAA, and TCM supplementation would more effectively support exercise adaptation as evaluated through blood ammonia concentration and aerobic capacity than combined extra- (ALK) cellular buffering agent, BCAA and TCM treatment.

## Methods

### Participants

**Fig. 1:**
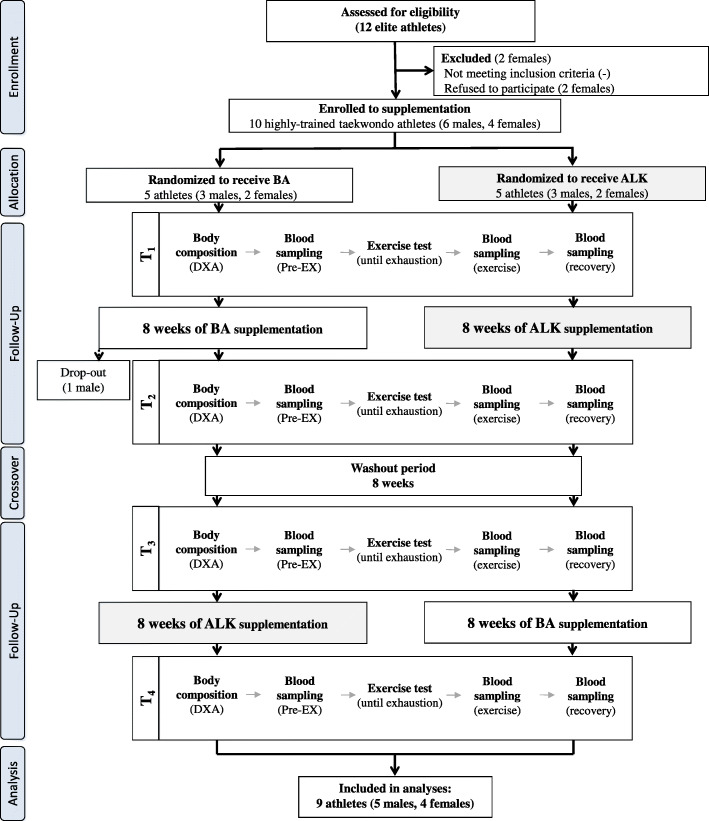
A flow chart of the study design. Abbreviations: *ALK* multi-ingredient extra-cellular buffering supplement, combined with branched-chain amino acids and creatine malate; *BA* multi-ingredient intra-cellular buffering supplement, combined with branched-chain amino acids and creatine malate; *DXA* Dual X-ray Absorptiometry; *Pre-EX* pre-exercise values; *T*_*1*_*-T*_*4*_ the order of laboratory visits and tests (form 1st to 4th visit)

Twelve taekwondo athletes were initially enrolled in this study. Eventually, 9 participants (5 male and 4 female) completed the entire study protocol and were included in analyses. The participants were members of the Polish national team meeting specific criteria, i.e. highest level of training status, physical capacity, performance, and technical skills. All of the participants were black belt holders and medalists in ranked competitions (international open tournaments, Universiade, European, and world championships). One athlete took part in the Olympic Games. The inclusion criteria were age from 18 to 35 years, good health, a valid medical certificate confirming the ability to compete and practice sports, and at least 5 years of professional taekwondo training experience. The gender-related impact on the cross-over design of the study was considered negligible. Exclusion criteria were: current smoking or illicit drug use, alcohol consumption greater than 1–2 drinks/week, and dietary supplements beyond those recommended within this study. For females, additional exclusion criteria were being pregnant or planning to become pregnant during the study. Basic training characteristics were also monitored before and during the whole study protocol (initial period, first treatment period, washout, and second treatment period; Table S1). All athletes maintained a high-intensity exercise regime characteristic of taekwondo training and combat during the whole treatment and washout periods. Importantly, each treatment period was preceded by a similar standard training period. Furthermore, all athletes declared that they did not introduce any changes in their lifestyles, especially nutrition, and that they did not use any medications and supplements with potential ergogenic effects other than those supplied by the authors of this study. Dietary records were being performed for two days every second week (current quotation) to ensure that the athletes had not changed their dietary habits during the whole supplementation period. The team member responsible for the diet analysis randomly informed the subjects about the need to start recording their two-day menus. Nutritional data was collected using notes on mobile phones and taking pictures of the meals consumed, which were then sent to the person analyzing the data. Dietary assessment was carried out using the Dietetyk-2 (Jumar, Poznań, Poland) software package following the previously described procedures used in our studies, both including supplementation and nutritional interventions, and weight management [8, 27–29]. The national team coaches enabled the confirmation of the required inclusion criteria declared by the participants. They also supported the control of training and supplementation compliance by monitoring the empty supplement containers, athletes’ diaries, and periodic personal observations of supplementation use by athletes. The project was approved by the Ethics Committee at the Poznan University of Medical Sciences (decision No. 143/15 of 5 February 2015) and was performed according to the ethical standards laid down in the Declaration of Helsinki. Each subject was informed of the testing procedure, purpose, and risks and submitted her/his written consent to participate. The study had been conducted for 1 year, from December 2015 to December 2016. The study complies with the CONSORT Statement for randomized trials as shown in Fig. 1.

### Experimental Design

#### Supplementation

The effect of supplementation was assessed in a randomized crossover double-blind trial. The primary outcomes were changes in total blood ammonia concentration (NH_3_), a specific marker of training adaptation, and aerobic capacity as assessed during an incremental treadmill test until exhaustion. In our previous similar study [8], we assessed the same effects in two extreme cases of exercise performance in highly-trained sprinters vs. endurance athletes. In this study, it was particularly essential to assess the effect of similar supplementation protocol in disciplines with mixed energetics. Of twelve participants, 1 male and 2 females did not complete the study either due to their refusal to participate or resignation without explanation (Fig. 1). After qualifying for the experiment, the athletes were subjected to a randomization procedure and assigned either to the group receiving (i) BA (Beta-Alanine Carno Rush Mega Tabs®, BCAA Mega Caps®, TCM Mega Caps®, and placebo (maltodextrin, PLA) instead of Alkagen™) or (ii) ALK (Alkagen™, BCAA Mega Caps®, TCM Mega Caps®, and PLA instead of Beta-Alanine Carno Rush Mega Tabs®) preparations. The random allocation sequence and assigning participants to supplementation with preparations with specific codes was performed by an impartial scientist who was not a member of the research team. The experimental procedure included an 8-week BA and ALK supplementation. After this period, an 8-week washout period was introduced. The next step was a crossover exchange of the preparations. The 8-week washout and 8-week supplementation periods were established, similar to other studies [30, 31].

Detailed supplementation characteristics are presented in Table 1. During each series of supplementation, all athletes were given the equivalent quantitative doses of about 0.2 g·kg_FFM_^− 1^ BCAA (11.0 ± 2.2 g·day^− 1^ BCAA; BCAA Mega Caps®; 1100 mg·cap^− 1^) and about 0.05 g·kg_FFM_^− 1^ creatine malate (2.8 ± 0.6 g·day^− 1^ TCM; TCM Mega Caps®; 1100 mg·cap^− 1^). In the BA group, the following preparations were also administered depending on the study phase: 5 capsules of Beta-Alanine Carno Rush Mega Tabs® (containing βA (1000 mg·cap^− 1^), sodium citrate (150 mg·cap^− 1^), L-histidine HCl (500 mg·cap^− 1^), and vitamin B_6_ (0.35 mg·cap^− 1^)), and PLA (maltodextrin) imitating Alkagen™ alkalizing formulation; the specified doses indicated in Table 1. Concurrently, in the ALK group, the following preparations were administered: about 0.2 g·kg_FFM_^− 1^ Alkagen™ (11.0 ± 2.2 g·day^− 1^ ALK) alkalizing preparation (containing SB (375 mg·cap^− 1^), potassium bicarbonate (375 mg·cap^− 1^), calcium phosphate (150 mg·cap^− 1^), potassium citrate (125 mg·cap^− 1^), magnesium citrate (125 mg·cap^− 1^), calcium citrate (90 mg·cap^− 1^), magnesium oxide (30 mg·cap^− 1^), zinc (0.375 mg·cap^− 1^)), and a PLA (maltodextrin instead of a Beta-Alanine Carno Rush Mega Tabs); the specified doses indicated in Table 1. The number of capsules (Alkagen, BCAA, TCM) was adjusted to match the prescribed dose in g·kg_FFM_^− 1^ as close as possible. The preparations were administered in 4 split doses. If one training session a day was foreseen, the split doses were administered at the following times: upon awakening, 60 min before the training session, immediately after the training session, and before sleep. If two training sessions a day were scheduled the times were as follows: upon awakening, 60 min before each training session, and before sleep.

All products, including PLA preparations, were prepared by one manufacturer (Olimp Laboratories, Dębica, Poland). Preparations were coded, making it impossible to identify and assign the same preparation twice to the same subject.

**Table 1:** Detailed supplementation characteristics of the examined athletes

BA			ALK		
Ingredient	Total daily doses		Ingredient	Total daily doses	
	Individual dose	Whole group (g·day^− 1^)		Individual dose	Whole group (g·day^− 1^)
		Mean ± SD			Mean ± SD
BCAA	0.20 g·kg_FFM_^−1^	11.0 ± 2.2	BCAA	0.2 g·kg_FFM_^−1^	11.0 ± 2.2
TCM	0.05 g·kg_FFM_^−1^	2.8 ± 0.6	TCM	0.05 g·kg_FFM_^−1^	2.8 ± 0.6
Βeta-alanine	5.0 g	5.0	Sodium bicarbonate	0.07 g·kg_FFM_^−1^	3.9 ± 0.7
Sodium citrate	0.75 g	0.75	Potassium bicarbonate	0.07 g·kg_FFM_^−1^	3.9 ± 0.7
L-histidine HCl	2.5 g	2.5	Calcium phosphate	0.03 g·kg_FFM_^−1^	1.7 ± 0.3
Vitamin B_6_	1.75 mg	0.00175	Potassium citrate	0.025 g·kg_FFM_^−1^	1.4 ± 0.3
			Magnesium citrate	0.025 g·kg_FFM_^−1^	1.4 ± 0.3
			Calcium citrate	0.018 g·kg_FFM_^−1^	1.0 ± 0.2
			Magnesium oxide	0.006 g·kg_FFM_^−1^	0.3 ± 0.1

Abbreviations: *ALK *multi-ingredient extra-cellular buffering supplement, combined with branched-chain amino acids and creatine malate; *BA *multi-ingredient intra-cellular buffering supplement, combined with branched-chain amino acids and creatine malate; *BCAA *branched-chain amino acids; *FFM *fat-free mass; *SD *standard deviation; *TCM *creatine malate

#### Laboratory Visits

The study protocol included four visits to the Human Movement Laboratory “_L_A_B_THLETICS” of the Department of Athletics, Strength and Conditioning at the Poznan University of Physical Education, Poznań, Poland (Fig. 1). Subjects were instructed not to participate in any high-intensity or long-duration training sessions at least 24 h before testing. All measurements were performed in the morning, 3 h after a light breakfast (no coffee or tea). At the start, subjects underwent body composition analysis. Afterward, an incremental treadmill test until volitional exhaustion was performed. During all measurements, the ambient temperature remained at 20‒21^o^C. The subjects were familiar with the tests and procedures used as they had participated in some previous research projects.

##### Anthropometry and Body Composition

Body mass (kg) and height (cm) were measured using a digital stadiometer (SECA 285, Hamburg, Germany). Body mass index (BMI) was calculated by dividing body mass by height squared. The Dual X-ray Absorptiometry (DXA) method, utilizing the Lunar Prodigy Pro device (GE Healthcare, Madison, Wisconsin, USA) and enCORE v. 16 SP1 software, was used for body composition assessment. During the examination, subjects only wore their undergarments, without jewelry and metal objects to minimize measurement error. The measurements were carried out according to the standardized scanning protocol as recommended by Nana et al. [32].

##### Cardiorespiratory Test

An exercise test on the Pulsar mechanical treadmill (H/P Cosmos Sports & Medical GmbH, Nussdorf-Traunstein, Germany) was performed to determine maximal oxygen uptake (V̇O_2max_). The initial speed was set at 4 km·h^−1^ and increased after 3 min to 8 km·h^−1^. After that point, treadmill speed progressively increased by 2 km·h^−1^ every 3 min until volitional exhaustion. After the speed of 10 km·h^−1^ was reached, venous blood samples were drawn at the end of each 3-min stage during pauses of up to 20 s. Minute ventilation (V̇E), oxygen uptake (V̇O_2_), carbon dioxide production, respiratory exchange ratio (RER), and other respiratory parameters were measured (breath by breath) by the Metamax 3B R2 ergospirometer and analyzed using MetasoftStudio v. 5.1.0 Software (Cortex Biophysik, Leipzig, Germany). Heart rate (HR) was monitored using the Bluetooth Smart H6 heart rate monitor (Polar Electro Oy, Kempele, Finland). The V̇O_2_max and corresponding exercise variables were considered to be reached when at least three of the following criteria were met: (1) a plateau in V̇O_2_ despite an increase in speed and minute ventilation; (2) blood lactate concentration at exhaustion ≥ 9 mmol∙L^−1^, (3) respiratory exchange ratio ≥ 1.10, and (4) heart rate ≥ 95 % of maximum (based on previous measurements) [33]. Also, V̇O_2_, V̇E, HR, and RER at the ventilatory threshold were determined based on the ventilatory equivalents and partial pressures of oxygen and carbon dioxide.

##### Blood Sampling

A catheter (BD Venflon Pro 1.3 × 32 mm, Helsingborg, Sweden) patent with isotonic saline (0.9 % NaCl) was placed in the antecubital vein. Blood samples were taken into two monovettes (S-Monovette 2.7 ml KE, Sarstedt, Nümbrecht, Germany), one with a lithium anticoagulant (heparin) for lactate and NH_3_ assay and another containing the EDTA anticoagulant for hematological measurements.

##### Lactate and Ammonia

To determine lactate concentration (LA), 20 µl of whole blood was placed into a capillary and placed in the Biosen C-line device (EKF diagnostic GmbH, Barleben, Germany). To determine total blood ammonia concentration, the PocketChem BA PA-4140 device (Arkray, Kyoto, Japan) was used with measuring range and accuracy (CV) of 8‒285 µmol·L^−1^ and 2.3 %, respectively. Immediately after drawing blood, a sample of 20 µl was placed on the test strip (Ammonia Test Kit II, Arkay, Kyoto, Japan) using a pipette. Ammonium ions in the sample were converted into the gaseous form that reacted with an indicator layer to change its color. The light of a wavelength of 635 nm, reflected by the indicator layer, was used to determine the level of color indicative of ammonia concentration.

##### Hematological Measurements

Ten µl of blood were analyzed in the automated Mythic®18 analyzer (Orphée, Geneva, Switzerland) to determine concentrations of white blood cells (WBC), lymphocytes (Lym), monocytes (Mon), granulocyte (Gra), red blood cells (RBC), hemoglobin (Hb), and hematocrit (Ht).

### Statistical analysis

Stratified randomization was performed with fat-free mass (FFM) being a prognostic variable as described previously [8, 30, 34, 35]. The results are presented as means ± standard deviation (and 95 % confidence intervals). The normality of data was tested using the Shapiro-Wilk test. If the distribution was not normal, the Box-Cox transformation was applied. Data were analyzed using repeated measures within-between interaction analysis of variance (ANOVA) with the inclusion of experimental supplementation order (BA first or ALK first), which allowed for the elimination of the potential carryover effect. The analysis also included factors independent of time: treatment (BA/ALK) x period (Before/After). Post hoc analysis was done using Bonferroni correction. If the sphericity assumption was violated, the Greenhouse-Geisser and the Huynh-Feldt corrections were performed. The sample size was estimated a priori, assuming that the effect size of supplementation type on ammonia blood concentration was 0.42 (partial eta-squared) as shown in our previous study [8]. Using an α-level of 0.05, a power (1 – β) of 0.80, it was calculated that at least 8 participants in each supplementation group would be needed to detect a significant change or differences in the variables analyzed (G*Power; Heinrich-Heine-Universität, Düsseldorf, Germany). To compare the anthropometric traits, training history, and diet characteristics, independent samples t-tests or Mann–Whitney U tests were performed, depending on data distribution (parametric or nonparametric, respectively). Statistical significance was set at *p* < 0.05 and data were analyzed using the Statistica 12 software package (StatSoft Inc., Tulsa, OK, USA).

## Results

### Baseline characteristics

The baseline characteristics of the studied taekwondo athletes are presented in Table 2. After randomization, there were no differences between the groups.

**Table 2:** Baseline anthropometric, training, and diet characteristics of the examined athletes

		BA→ALK group			ALK→BA group			BA→ALK vs. ALK→BA group
		Mean	±	SD	Mean	±	SD	*p-value*
N		5			4			-
Age	(years)	21	±	3	19	±	3	0.27
Body height	(cm)	181	±	6	179	±	10	0.60
Body mass	(kg)	68.1	±	1.2	70.2	±	14.5	0.75
Fat mass	(kg)	13.8	±	5.6	14.5	±	3.6	1.00
Fat-free mass	(kg)	54.2	±	5.5	56.2	±	16.9	0.81
V̇O_2max_	(L·min^−1^)	3.65	±	0.67	3.68	±	1.09	0.95
Training experience	(years)	8	±	1	9	±	3	0.52
Energy intake	(kcal·day^−1^)	2343	±	492	2796	±	472	0.20
	(kcal·kg^−1^)	35.1	±	7.3	41.6	±	14.0	0.39
Protein intake	(g·day^−1^)	109	±	19	111	±	15	0.86
	(g·kg^−1^)	1.6	±	0.3	1.6	±	0.5	0.71
	(% of energy)	18.9	±	3.1	16.2	±	2.9	0.21
Fat intake	(g·day^−1^)	71	±	27	106	±	42	0.17
	(g·kg^−1^)	1.1	±	0.4	1.6	±	0.7	0.21
	(% of energy)	26.6	±	6.1	33.1	±	10.6	0.28
Carbohydrate intake	(g·day^−1^)	338	±	60	379	±	57	0.33
	(g·kg^−1^)	5.1	±	0.9	5.7	±	2.0	0.56
	(% of energy)	58.1	±	3.4	55.0	±	9.1	0.51

Values are expressed as means ± standard deviation (SD). Abbreviations: *ALK *multi-ingredient extra-cellular buffering supplement; *ALK→BA group *the order of implementing the supplementation, 1st ALK, 2nd BA after wash-out; *BA *multi-ingredient intra-cellular buffering supplement; *BA→ALK group *the order of implementing the supplementation, 1st BA, 2nd ALK after wash-out; *V̇O*_*2max*_ maximal oxygen uptake

### Body composition

No significant body mass and composition differences (*p* > 0.05) were revealed between BA and ALK groups and before versus after supplementation (Table 3).

**Table 3:** Body mass and composition in taekwondo athletes before and after supplementation

			BA						ALK					
			Mean	±	SD	95% CI			Mean	±	SD	95% CI		
Body mass	(kg)	Before	68.0	±	9.9	60.4	-	75.6	67.0	±	9.0	60.1	-	73.9
		After	69.1	±	9.3	62.0	-	76.3	69.0	±	8.7	62.3	-	75.6
BMI	(kg∙m^−2^)	Before	21.1	±	2.3	19.3	-	22.8	20.7	±	2.1	19.1	-	22.3
		After	21.4	±	2.1	19.8	-	23.1	21.4	±	2.1	19.7	-	23.0
Total Fat Mass	(kg)	Before	12.6	±	4.0	9.5	-	15.8	12.6	±	3.9	9.6	-	15.6
		After	13.8	±	3.9	10.8	-	16.8	14.1	±	4.6	10.5	-	17.6
Total Fat-Free Mass	(kg)	Before	55.5	±	12.2	46.1	-	64.8	54.9	±	11.2	46.3	-	63.5
		After	55.8	±	11.2	47.2	-	64.4	55.4	±	11.4	46.6	-	64.1

Data are the means ± standard deviation (SD) and 95% confidence intervals (CI). Abbreviations: *After* after supplementation; *ALK* multi-ingredient extra-cellular buffering supplement, combined with branched-chain amino acids and creatine malate; *BA* multi-ingredient intra-cellular buffering supplement, combined with branched-chain amino acids and creatine malate; *Before* before supplementation; *BMI* body mass index.

### Pre-exercise and exercise cardiorespiratory indices

No significant differences in aerobic capacity indices (*p* > 0.05) were found between/within groups and before/after supplementation (Table 4).

**Table 4:** Pre-exercise and exercise cardiorespiratory variables in taekwondo athletes before and after supplementation

			BA						ALK					
			Mean	±	SD	95% CI			Mean	±	SD	95% CI		
V̇E_Pre-EX_	(L∙min^−1^)	Before	14.0	±	2.5	12.1	-	16.0	14.1	±	2.0	12.6	-	15.7
		After	13.9	±	2.8	11.7	-	16.0	15.9	±	2.8	13.7	-	18.0
HR_Pre-EX_	(bpm)	Before	83	±	13	73	-	93	80	±	13	70	-	90
		After	83	±	12	73	-	92	85	±	13	75	-	95
V̇O_2Pre-EX_	(L∙min^−1^)	Before	0.49	±	0.09	0.42	-	0.56	0.47	±	0.07	0.42	-	0.53
		After	0.48	±	0.07	0.43	-	0.54	0.56	±	0.11	0.47	-	0.65
RER_Pre-EX_		Before	0.79	±	0.04	0.76	-	0.83	0.83	±	0.05	0.79	-	0.87
		After	0.81	±	0.08	0.75	-	0.88	0.81	±	0.08	0.75	-	0.86
V̇E_VT_	(L∙min^−1^)	Before	68.7	±	20.5	53.0	-	84.5	67.1	±	19.7	52.0	-	82.3
		After	65.9	±	17.5	52.4	-	79.4	68.0	±	17.8	54.2	-	81.7
HR_VT_	(bpm)	Before	161	±	15	150	-	173	158	±	15	147	-	170
		After	159	±	15	148	-	171	161	±	18	147	-	174
V̇O_2VT_	(L∙min^−1^)	Before	2.55	±	0.68	2.03	-	3.08	2.59	±	0.70	2.05	-	3.13
		After	2.56	±	0.58	2.11	-	3.01	2.64	±	0.59	2.18	-	3.09
RER_VT_		Before	0.90	±	0.05	0.86	-	0.94	0.91	±	0.04	0.88	-	0.95
		After	0.89	±	0.08	0.83	-	0.96	0.89	±	0.05	0.85	-	0.93
V̇E_max_	(L∙min^−1^)	Before	129	±	30	106	-	152	129	±	31	106	-	153
		After	127	±	31	104	-	151	129	±	30	106	-	152
HR_max_	(bpm)	Before	189	±	14	178	-	200	192	±	11	183	-	200
		After	193	±	12	184	-	202	193	±	10	186	-	201
V̇O_2max_	(L∙min^−1^)	Before	3.64	±	0.84	2.99	-	4.29	3.54	±	0.77	2.95	-	4.14
		After	3.66	±	0.82	3.03	-	4.29	3.68	±	0.86	3.02	-	4.33
RER_max_		Before	1.10	±	0.05	1.06	-	1.13	1.10	±	0.03	1.07	-	1.13
		After	1.06	±	0.07	1.01	-	1.11	1.09	±	0.06	1.05	-	1.14

Data are the means ± standard deviation (SD) and 95% confidence intervals (CI). Abbreviations: *After* after supplementation; *ALK* multi-ingredient extra-cellular buffering supplement, combined with branched-chain amino acids and creatine malate; *BA* multi-ingredient intra-cellular buffering supplement, combined with branched-chain amino acids and creatine malate; *Before* before supplementation; *HR* heart rate; *max* maximum values; *Pre-EX* pre-exercise values; *RER* respiratory exchange ratio; *V̇E* minute ventilation; *V̇O*_*2*_ oxygen uptake; *VT* ventilatory threshold. No significant differences reported (*p* > 0.05).

### Blood biomarkers

**Fig. 2:**
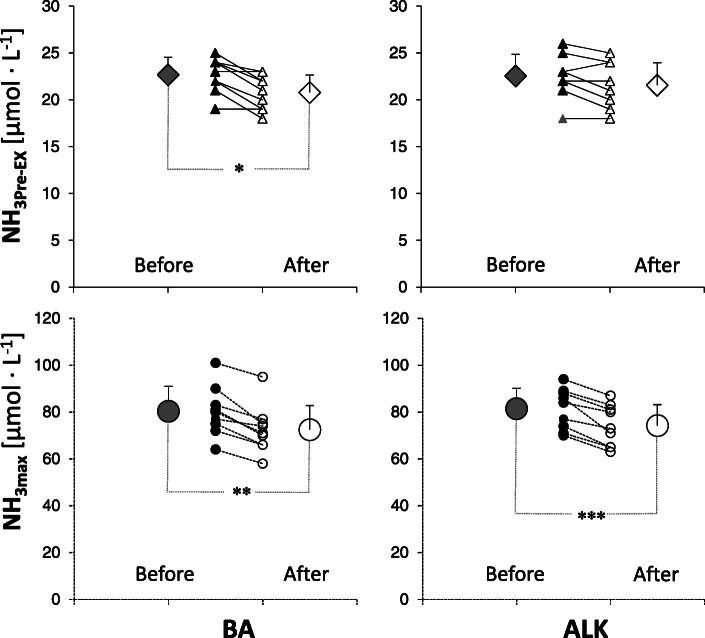
Blood ammonia (NH_3_) concentration during the post-exercise recovery before and after supplementation. Data are the means ± standard deviation (SD) and individual results. *,**,***: significant difference between “Before” and “After” values (* *p* = 0.006, ** *p* = 0.013, *** *p* = 0.027). Abbreviations: *After* after supplementation; *ALK* multi-ingredient extra-cellular buffering supplement, combined with branched-chain amino acids and creatine malate; *BA* multi-ingredient intra-cellular buffering supplement, combined with branched-chain amino acids and creatine malate; *Before* before supplementation; *max* maximum value after exercise; *Pre-EX* pre-exercise values

The concentration of two blood biomarkers and hematological parameters is given in Table 5; Fig. 2. There were no significant differences in BA and ALK groups before and after supplements administration, except NH_3_ concentration. In the BA group, pre-exercise (NH_3Pre−EX_) and maximal post-exercise (NH_3max_) ammonia concentration decreased significantly after supplementation protocols. In the ALK group, NH_3max_ concentration decreased significantly after the supplementation period (Fig. 2).

**Table 5:** The level of exercise blood biomarkers and hematological parameters in taekwondo athletes before and after supplementation (pre-exercise, maximum, and recovery values)

			BA						ALK					
			Mean	±	SD	95% CI			Mean	±	SD	95% CI		
LA_Pre-EX_	(mmol∙L^−1^)	Before	1.07	±	0.19	0.92	-	1.21	1.18	±	0.27	0.97	-	1.39
		After	1.13	±	0.26	0.93	-	1.33	1.10	±	0.30	0.88	-	1.33
LA_max_	(mmol∙L^−1^)	Before	10.9	±	1.6	9.7	-	12.1	10.9	±	2.1	9.3	-	12.5
		After	10.2	±	1.4	9.1	-	11.3	10.7	±	1.9	9.2	-	12.2
LA_R5_	(mmol∙L^−1^)	Before	10.33	±	1.64	9.07	-	11.59	9.99	±	1.50	8.83	-	11.14
		After	9.57	±	1.65	8.31	-	10.84	9.60	±	1.55	8.41	-	10.79
LA_R20_	(mmol∙L^−1^)	Before	6.37	±	1.77	5.01	-	7.73	6.27	±	1.66	4.99	-	7.54
		After	5.80	±	1.44	4.69	-	6.90	5.91	±	1.60	4.68	-	7.14
LA_R30_	(mmol∙L^−1^)	Before	4.63	±	1.58	3.42	-	5.85	4.21	±	1.51	3.05	-	5.36
		After	4.13	±	1.19	3.21	-	5.04	3.93	±	1.33	2.91	-	4.96
NH_3Pre-EX_	(μmol∙L^−1^)	Before	22.7	±	1.9	21.2	-	24.1	22.6	±	2.3	20.8	-	24.3
		After	20.8	±	1.9*	19.4	-	22.2	21.6	±	2.4	19.7	-	23.4
NH_3max_	(μmol∙L^−1^)	Before	80.3	±	10.6	72.2	-	88.5	81.4	±	8.7	74.8	-	88.1
		After	72.4	±	10.2**	64.6	-	80.3	74.2	±	8.9 ***	67.4	-	81.0
NH_3R5_	(μmol∙L^−1^)	Before	66.4	±	15.9	54.2	-	78.7	69.2	±	11.4	60.4	-	78.0
		After	61.1	±	17.8	47.4	-	74.8	61.8	±	8.7	55.1	-	68.5
NH_3R20_	(μmol L^−1^)	Before	48.2	±	11.5	39.4	-	57.1	48.8	±	6.8	43.6	-	54.0
		After	43.9	±	12.4	34.3	-	53.4	44.4	±	6.3	39.6	-	49.3
NH_3R30_	(μmol∙L^−1^)	Before	38.8	±	6.5	33.8	-	43.8	37.8	±	4.4	34.4	-	41.1
		After	34.8	±	8.5	28.2	-	41.3	33.6	±	4.6	30.0	-	37.1
WBC_Pre-EX_	(x10^9^∙L^−1^)	Before	5.88	±	1.32	4.86	-	6.89	5.76	±	0.98	5.00	-	6.51
		After	5.27	±	0.98	4.51	-	6.02	5.06	±	0.80	4.44	-	5.67
Lym_Pre-EX_	(x10^9^∙L^−1^)	Before	1.74	±	0.24	1.56	-	1.93	1.76	±	0.36	1.48	-	2.03
		After	1.91	±	0.19	1.77	-	2.06	1.86	±	0.40	1.55	-	2.17
Mon_Pre-EX_	(x10^9^∙L^−1^)	Before	0.30	±	0.10	0.22	-	0.38	0.41	±	0.15	0.30	-	0.52
		After	0.38	±	0.15	0.26	-	0.49	0.39	±	0.13	0.29	-	0.49
Gra_Pre-EX_	(x10^9^∙L^−1^)	Before	3.81	±	1.28	2.83	-	4.79	3.60	±	0.79	2.99	-	4.21
		After	2.98	±	0.82	2.35	-	3.60	2.81	±	0.58	2.37	-	3.26
RBC_Pre-EX_	(x10^12^∙L^−1^)	Before	4.68	±	0.50	4.29	-	5.06	4.96	±	0.53	4.55	-	5.38
		After	4.60	±	0.39	4.30	-	4.90	4.65	±	0.38	4.36	-	4.95
Hb_Pre-EX_	(mmol∙L^−1^)	Before	8.60	±	0.86	7.94	-	9.27	8.78	±	0.64	8.28	-	9.27
		After	8.47	±	0.84	7.83	-	9.12	8.36	±	0.43	8.03	-	8.69
Ht_Pre-EX_	(L∙L^−1^)	Before	0.40	±	0.04	0.37	-	0.43	0.41	±	0.03	0.39	-	0.44
		After	0.40	±	0.03	0.37	-	0.42	0.39	±	0.02	0.38	-	0.41

Data are mean ± standard deviation (SD) and 95% confidence intervals (CI). *,**,***: significant difference between “Before” and “After” values (* *p*=0.006, ** *p*= 0.013, *** *p*=0.027). Abbreviations: *After* after supplementation; *ALK* multi-ingredient extra-cellular buffering supplement, combined with branched-chain amino acids and creatine malate; *BA* multi-ingredient intra-cellular buffering supplement, combined with branched-chain amino acids and creatine malate; *Before* before supplementation; *Gra* granulocytes; *Hb* hemoglobin; *Ht* hematocrit; *LA* lactate; *Lym* lymphocytes; *Mon* monocytes; *NH*_*3*_ ammonia; *Pre-EX* pre-exercise values; *R* post-exercise recovery period (5, 20, 30 = minute of recovery); *RBC* red blood cells; *TCM* creatine malate; *WBC* white blood cells

## Discussion

In this study, elite taekwondo athletes were supplemented with BA or ALK combined with BCAA and TCM administration during an 8-week training period (description in Table S1). We did not observe any significant changes in body mass and composition neither in the BA nor ALK group. Despite many studies suggesting the effect of such preparations on respiratory and aerobic capacity, we did not observe any major impact. Also, we did not reveal any significant changes in hematological parameters. As far as exercise blood biomarkers are concerned, the only change we observed was the statistically significant decrease in NH_3max_ concentration after administration of both BA and ALK supplementation combined with BCAA and TCM.

Supplements and their doses used in this study were chosen after evaluating the available scientific literature and determining the efficacy and safety of these substances [12]. Moreover, elite athletes participating in our study were healthy and particularly in need of taking these supplements, mainly for proper recovery, training adaptation, or avoidance of health consequences.

No significant differences in body mass and composition are in agreement with the findings of Kendrick et al. [36] who revealed that a 10-week resistance training combined with βA did not significantly change body fat percentage. Also, Smith et al. [37] showed no significant changes in body mass, fat mass, and fat percentage, but they reported significant increases in lean body mass in a βA group after high-intensity interval training. A study on highly trained sprinters showed increases in total fat-free mass after βA supplementation (combined with BCAA and TCM) [8]. In contrast, Kern and Robinson [38] showed no significant changes in lean body and fat mass in trained football players and wrestlers supplemented with βA. However, Hoffman et al. [39] reported a greater increase in lean body mass and a decrease in the percentage of body fat after βA and CR supplementation compared to CR alone in strength/power athletes, even though no between-protocol differences in total body and fat mass were observed. It seems that the gain in lean body mass after βA treatment, reported in some studies [37, 39], can be partly explained by minimizing the exercise-induced muscle acidification and is related to the preparedness for increases in training volume and/or improved muscle metabolism. We have not found any studies monitoring body composition changes in response to SB treatment in highly trained athletes. Our study suggests that supplementation of alkaline agents during several weeks does not affect body composition in striking combat sports contestants, specifically taekwondo athletes.

We found no significant differences in cardiorespiratory exercise indices after any of the supplementation protocols. It must be stressed that taekwondo training and combat are characterized by mixed energetics. In simulated taekwondo combats, the contribution of aerobic, anaerobic phosphagen, and glycolytic energy systems is 66 %, 30 %, and 4 %, respectively, with a probable shift towards anaerobic energy sources during real tournaments [40, 41]. Our results are in agreement with the study by Baguet et al. [42] that found no effect on V̇O_2peak_ after βA supplementation in physically active males. It also seems that high-intensity interval training (HIIT) better supports aerobic capacity gains than moderate-intensity exercise. Although taekwondo training is largely based on specific HIIT, in our opinion the aerobic adaptation changes could not be detectable in elite athletes who took part in our study. Their aerobic capacity was already at a high level (optimal for sports discipline they practice) and it was hard to expect further improvements. Lopes-Silva et al. [43] conducted a simulated combat study on 9 male taekwondo athletes ingesting SB and demonstrated no significant differences in HR and V̇O_2_ between the SB group and placebo, either. In the latest crossover study including elite sprinters and endurance athletes, respiratory and aerobic capacity indices also remained unchanged after BA and ALK supplementation [8], which further confirms the results we obtained from the taekwondo athletes.

Unlike competitive athletes, recreationally active people usually noticeably improve their aerobic capacity (peak V̇O_2_) if combined supplementation with βA and HIIT is administered [37]. Zoeller et al. [44] measured the effect of supplementation with βA in combination with CR on aerobic exercise performance in 55 men and demonstrated a significant improvement in V̇O_2max_ and V̇O_2_ at the ventilatory threshold with combined supplementation group compared to βA or CR alone. In SB supplementation studies, progressive-dose SB ingestion improved CrossFit-specific performance and delayed ventilatory threshold occurrence [30]. Studies involving recreationally active people also proved the effect of SB supplementation on cardiorespiratory exercise indices [45, 46]. Summarizing these aspects, training status and sports performance level may have a crucial impact on the effect of agents supporting buffer capacity. This effect can be less visible or not visible at all in competitive elite athletes representing a high level of exercise adaptation.

Earlier studies showed significant changes in blood lactate concentration after supplementation with alkaline agents. Artioli et al. [47], Carr et al. [24], and Saunders et al. [48] found that acute SB supplementation (0.3 g·kg^−1^ before exercise) resulted in higher lactate concentration in experienced judo competitors, resistance-trained males, and recreationally active men, respectively. Other studies including athletes [43, 49] also showed that peak blood lactate concentration increased after acute SB administration (0.3 g·kg^−1^ before exercise). Likewise, studies on recreationally active people [45, 46, 50] resulted in higher blood lactate concentration compared to placebo. It was suggested that increased blood lactate after SB ingestion was observed in individuals who improved their exercise capacity [48]. SB ingestion increases lactate concentration due to increased efflux of lactate and H^+^ from muscles into the extracellular fluid [43]. It seems that the differences between our observations and other research result from different supplementation protocols (chronic vs. acute supplementation). The available data indicate that acute treatment with a higher dose may be more effective than chronic supply in divided doses [8, 27, 30]. In our study, the doses of SB might have been relatively too low to produce significant effects.

Insignificant changes in lactate concentration in this study could result from the specificity of the progressive treadmill test that diverged from workload during typical taekwondo training sessions or tournament combats. Kern and Robinson [38] showed only minor changes in blood lactate concentrations in the placebo and βA-supplemented groups (small increases in trained football players and small decreases in wrestlers). Kratz et al. [10] found that βA administration in judokas resulted in significant increases in lactate concentration. As mentioned above, this may suggest that, similarly to SB, βA improves the ability to clear or tolerate higher muscle acidification, depending on exercise specificity, allowing athletes to exercise at a higher intensity or over a longer period.

In our study, the only significant change was detected for NH_3_ after both BA (pre-exercise and maximum concentrations) and ALK (maximum concentration) supplementation. To our knowledge, this is the first study to demonstrate such an effect on NH_3_ concentration in highly trained combat sports athletes. There is relatively little literature data, mainly concerning endurance disciplines, on the connection between ergogenic supplementation and NH_3_ concentration in athletes. The decreased maximum post-exercise blood ammonia concentration observed in our study may be metabolically important since previous reports revealed that NH_3_ is a sensitive marker of the exercise-induced metabolic response to incremental exercise [51–53]. Hsueh et al. [54] indicated that combined supplementation with BCAA, arginine, and citrulline did not change pre- and post-exercise NH_3_ concentration. However, our previous study on the BA treatment combined with BCAA and TCM in international level sprinters and endurance athletes yielded a reverse result [8]. Some studies [55–57] suggest that the use of certain single ergogenic supplements may decrease exercise-induced hyperammonaemia. A smaller NH_3_ concentration might hinder the harmful influence of ammonia on protein synthesis and BCAA catabolism, supporting performance, e.g. through the suppression of central fatigue and motor coordination aggravation [51, 58, 59]. Furthermore, a lower post-exercise increase in NH_3_ concentration can be related to lower adenosine triphosphate depletion and slower glycolysis and glycogenolysis rate, especially in exercising skeletal muscles [53, 60]. In this current study, the change was statistically significant but its practical relevance may be questionable because no other measure we used supported the potential effect of NH_3_ reduction on better exercise economy, lower physiological cost of muscle work, reduced fatigue, or enhanced metabolic adaptation. This issue requires further research.

This study has some limitations. We cannot be sure that the athletes completely adhered to our recommendations. Also, other exercise-derived data than those concerning aerobic adaptation could help to better interpret the results (e.g. high-intensity anaerobic and discipline-specific tests). The duration of exercise in the high-intensity zone during the progressive test was probably too short to reveal more clear-cut changes in the supplementation-induced indices of exercise capacity. Future studies should focus on implementing high-intensity exercise tests. There was also no control/placebo group, however, only elite athletes had been involved in the study and it was not possible to apply a procedure in which athletes would only have to receive a placebo or not be supplemented at all. It is important to underline that it was not possible to conduct controlled studies on these athletes without their customary supplementation. Elite athletes could be only supplemented and tested during the specific period, taking into account long-term training and competition plans. The inclusion of athletes with lower training status would make the results incomparable between the groups. Probably, a larger sample size would give more pronounced results. The numbers of training sessions/hours and workout specificity fluctuated somewhat across the consecutive 8-week periods, however, the volume and intensity remained comparable, as previously described [37]. This, and the cross-over design, minimized the confounding effect of changes in training loads and allowed to isolate the effect of the administered supplementation. However, it should be noted that in our study training loads and some minor factors could not be fully controlled. Even if training loads would be the same across the 24 weeks, the athletes’ training status would gradually change due to physiological adaptation. On the other hand, our study provides insight into the actual effects of supplementation during the real training process, contrary to laboratory studies conducted in ‘artificial’ conditions, not adequate to typical competitive sports participation. As we described in the methods section, all athletes maintained taekwondo-specific high-intensity training before and during the entire study period. Finally, it is important to underline that the doses of the supplements could be not high enough to produce substantial changes in monitored indices. Nevertheless, the strength of our research is the participation of current elite athletes, supplementation during the real training process, group homogeneity, and application of the cross-over design study.

Many coaches and highly trained athletes are convinced that the lack of supplementation negatively affects physical performance and nutritional status. Consequently, combined supplementation is administered as a preventive measure during critical periods of the annual training cycle, i.e. when (i) athletes are loaded with many training units a day/week, (ii) they have very short and often sub-optimal recovery breaks between training sessions, or (iii) covering the high nutritional needs using a standard diet is difficult. Athletes customarily use multiple supplements intending to enhance their performance and enable participation in heavy training cycles. Our research is partly filling in the gap in evidence-based studies on the combined supplementation use among trained athletes, especially on buffering agents and other ergogenic supplements. The future challenge for scientists and medical staff (sports nutritionists, physiologists, and medical doctors) is the elaboration of practical and effective strategies to counteract the negative effects of the exercise-induced acid-base imbalance.

## Conclusions

In highly trained taekwondo athletes, both extra- and intracellular buffering enhancement resulting from an 8-week BA and ALK supplementation, respectively, combined with BCAA and TCM treatment, solely resulted in a modest reduction in total blood ammonia concentration at the exercise intensity corresponding to V̇O_2_max, however, without significant changes in body mass and composition, aerobic capacity, and hematological indices.

## Supplementary Information

**Additional file 1:** **Supplementary Table S1**. The number, duration, and average intensity (percentage of maximum heart rate) of training sessions and tournaments in tested taekwondo athletes before, during the supplementation and washout periods.

## Data Availability

The datasets used and/or analyzed during the current study are available from the corresponding author on request.

## Abbreviations

ALK: Multi-ingredient extra-cellular buffering supplement, combined with branched-chain amino acids and creatine malate
After: After supplementation
BA: Multi-ingredient intra-cellular buffering supplement, combined with branched-chain amino acids and creatine malate
BCAA: Branched-chain amino acids
Before: Before supplementation
BMI: Body mass index
βA: Beta-alanine
CI: Confidence intervals
CR: Creatine
CV: Measurement accuracy
DXA: Dual X-ray Absorptiometry
FFM: Fat-free mass
Gra: Granulocyte
Hb: Hemoglobin
HR: Heart rate
Ht: Hematocrit
LA: Lactate concentration
Lym: Lymphocytes
Mon: Monocytes
NH_3_: Total blood ammonia
PCr: Phosphocreatine
PLA: Placebo
Pre-EX: Pre-exercise values
R: Post-exercise recovery period
RBC: Red blood cells
RER: Respiratory exchange ratio
SB: Sodium bicarbonate
SD: Standard deviation
TCM: Creatine malate
V̇E: Minute ventilation
V̇O_2max_: Maximal oxygen uptake
V̇O_2_: Oxygen uptake
VT: Ventilatory threshold
WBC: White blood cells

## References

[1] Jarvis K, Woodward M, Debold EP, Walcott S (2018). Acidosis affects muscle contraction by slowing the rates myosin attaches to and detaches from actin. J Muscle Res Cell Motil.

[2] Robergs RA, Ghiasvand F, Parker D (2004). Biochemistry of exercise-induced metabolic acidosis. Am J Physiol Regul Integr Comp Physiol.

[3] Robergs RA. Exercise-induced metabolic acidosis: Where do the protons come from? Sportscience. 2001;5(2).

[4] van Meerhaeghe A, Velkeniers B (2005). Lactate production and exercise-induced metabolic acidosis: guilty or not guilty?. Eur Respir J.

[5] Chen S, Minegishi Y, Hasumura T, Shimotoyodome A, Ota N (2020). Involvement of ammonia metabolism in the improvement of endurance performance by tea catechins in mice. Sci Rep.

[6] Weiner ID, Verlander JW (2013). Renal ammonia metabolism and transport. Compr Physiol.

[7] Burke LM (2017). Practical issues in evidence-based use of performance supplements: supplement interactions, repeated use and individual responses. Sports Med.

[8] Durkalec-Michalski K, Kusy K, Ciekot-Sołtysiak M, Zieliński J (2019). The effect of beta-alanine versus alkaline agent supplementation combined with branched-chain amino acids and creatine malate in highly-trained sprinters and endurance athletes: a randomized double-blind crossover study. Nutrients.

[9] Chen I-F, Wu H-J, Chen C-Y, Chou K-M, Chang C-K (2016). Branched-chain amino acids, arginine, citrulline alleviate central fatigue after 3 simulated matches in taekwondo athletes: a randomized controlled trial. J Int Soc Sports Nutr.

[10] de Andrade Kratz C, de Salles Painelli V, de Andrade Nemezio KM, da Silva RP, Franchini E, Zagatto AM (2017). Beta-alanine supplementation enhances judo-related performance in highly-trained athletes. J Sci Med Sport.

[11] Manjarrez-Montes de Oca R, Farfán-González F, Camarillo-Romero S, Tlatempa-Sotelo P, Francisco-Argüelles C, Kormanowski A, et al. Effects of creatine supplementation in taekwondo practitioners. Nutr Hosp. 2013;28(2):391–9. 10.3305/nh.2013.28.2.6314 23822690

[12] Kerksick CM, Wilborn CD, Roberts MD, Smith-Ryan A, Kleiner SM, Jäger R (2018). ISSN exercise & sports nutrition review update: research & recommendations. J Int Soc Sports Nutr.

[13] Fedewa MV, Spencer SO, Williams TD, Becker ZE, Fuqua CA (2019). Effect of branched-chain amino acid supplementation on muscle soreness following exercise: a meta-analysis. Int J Vitam Nutr Res.

[14] Butts J, Jacobs B, Silvis M (2018). Creatine Use in Sports. Sports Health.

[15] Lancha Junior AH, Painelli V, de S, Saunders, Artioli B. GG. Nutritional strategies to modulate intracellular and extracellular buffering capacity during high-intensity exercise. Sports Med. 2015;45(Suppl 1):71–81. 10.1007/s40279-015-0397-5 PMC4672007 26553493

[16] Culbertson JY, Kreider RB, Greenwood M, Cooke M (2010). Effects of beta-alanine on muscle carnosine and exercise performance: a review of the current literature. Nutrients.

[17] Derave W, Özdemir MS, Harris RC, Pottier A, Reyngoudt H, Koppo K (2007). β-alanine supplementation augments muscle carnosine content and attenuates fatigue during repeated isokinetic contraction bouts in trained sprinters. J Appl Physiol (1985).

[18] Hill CA, Harris RC, Kim HJ, Harris BD, Sale C, Boobis LH (2007). Influence of β-alanine supplementation on skeletal muscle carnosine concentrations and high intensity cycling capacity. Amino Acids.

[19] Berti Zanella P, Donner Alves F, Guerini de Souza C (2017). Effects of beta-alanine supplementation on performance and muscle fatigue in athletes and non-athletes of different sports: a systematic review. Sports Med Phys Fitness.

[20] Heibel AB, Perim PHL, Oliveira LF, McNaughton LR, Saunders B (2018). Time to optimize supplementation: modifying factors influencing the individual responses to extracellular buffering agents. Front Nutr.

[21] Beaver WL, Wasserman K, Whipp BJ (1986). Bicarbonate buffering of lactic acid generated during exercise. J Appl Physiol (1985).

[22] de Oliveira LF, Saunders B, Yamaguchi G, Swinton P, Artioli GG (2020). Is individualization of sodium bicarbonate ingestion based on time to peak necessary?. Med Sci Sports Exerc.

[23] Grgic J, Garofolini A, Pickering C, Duncan MJ, Tinsley GM, Del Coso J (2020). Isolated effects of caffeine and sodium bicarbonate ingestion on performance in the Yo-Yo test: a systematic review and meta-analysis. J Sci Med Sport.

[24] Carr BM, Webster MJ, Boyd JC, Hudson GM, Scheett TP (2013). Sodium bicarbonate supplementation improves hypertrophy-type resistance exercise performance. Eur J Appl Physiol.

[25] Durkalec-Michalski K, Zawieja EE, Zawieja BE, Michałowska P, Podgórski T. The gender dependent influence of sodium bicarbonate supplementation on anaerobic power and specific performance in female and male wrestlers. Sci Rep. 2020;10(1):1878. 10.1038/s41598-020-57590-x PMC7002590 32024852

[26] Chou C-C, Sung Y-C, Davison G, Chen C-Y, Liao Y-H (2018). Short-term high-dose vitamin c and e supplementation attenuates muscle damage and inflammatory responses to repeated taekwondo competitions: a randomized placebo-controlled trial. Int J Med Sci.

[27] Durkalec-Michalski K, Zawieja EE, Podgórski T, Zawieja BE, Michałowska P, Łoniewski I (2018). The effect of a new sodium bicarbonate loading regimen on anaerobic capacity and wrestling performance. Nutrients.

[28] Durkalec-Michalski K, Nowaczyk PM, Siedzik K (2019). Effect of a four-week ketogenic diet on exercise metabolism in CrossFit-trained athletes. J Int Soc Sports Nutr.

[29] Durkalec-Michalski K, Zawieja EE, Zawieja BE, Jurkowska D, Buchowski MS, Jeszka J (2018). Effects of low versus moderate glycemic index diets on aerobic capacity in endurance runners: three-week randomized controlled crossover trial. Nutrients.

[30] Durkalec-Michalski K, Zawieja EE, Podgórski T, Łoniewski I, Zawieja BE, Warzybok M (2018). The effect of chronic progressive-dose sodium bicarbonate ingestion on CrossFit-like performance: A double-blind, randomized cross-over trial. PLoS ONE.

[31] Dolan E, Swinton PA, Painelli V, de S, Stephens Hemingway, Mazzolani B, Infante Smaira B. F, et al. A systematic risk assessment and meta-analysis on the use of oral β-alanine supplementation. Adv Nutr. 2019;10(3):452–63. 10.1093/advances/nmy115 PMC6520041 30980076

[32] Nana A, Slater GJ, Hopkins WG, Halson SL, Martin DT, West NP (2016). Importance of standardized DXA protocol for assessing physique changes in athletes. Int J Sport Nutr Exerc Metab.

[33] Edvardsen E, Hem E, Anderssen SA (2014). End criteria for reaching maximal oxygen uptake must be strict and adjusted to sex and age: a cross-sectional study. PLoS One.

[34] Trexler ET, Smith-Ryan AE, Stout JR, Hoffman JR, Wilborn CD, Sale C (2015). International society of sports nutrition position stand: beta-alanine. J Int Soc Sports Nutr.

[35] Durkalec-Michalski K, Jeszka J, Podgórski T. The Effect of a 12-week beta-hydroxy-beta-methylbutyrate (HMB) supplementation on highly-trained combat sports athletes: a randomised, double-blind, placebo-controlled crossover study. Nutrients. 2017;9(7):753. 10.3390/nu9070753 PMC5537867 28708126

[36] Kendrick IP, Harris RC, Kim HJ, Kim CK, Dang VH, Lam TQ (2008). The effects of 10 weeks of resistance training combined with beta-alanine supplementation on whole body strength, force production, muscular endurance and body composition. Amino Acids.

[37] Smith AE, Walter AA, Graef JL, Kendall KL, Moon JR, Lockwood CM (2009). Effects of beta-alanine supplementation and high-intensity interval training on endurance performance and body composition in men; a double-blind trial. J Int Soc Sports Nutr.

[38] Kern BD, Robinson TL (2011). Effects of β-alanine supplementation on performance and body composition in collegiate wrestlers and football players. J Strength Cond Res.

[39] Hoffman J, Ratamess N, Kang J, Mangine G, Faigenbaum A, Stout J (2006). Effect of creatine and beta-alanine supplementation on performance and endocrine responses in strength/power athletes. Int J Sport Nutr Exerc Metab.

[40] Janowski M, Zieliński J, Kusy K. Exercise response to real combat in elite taekwondo athletes before and after competition rule changes. J Strength Cond Res. 2019;10.1519/JSC.0000000000003110. 10.1519/JSC.0000000000003110 30844985

[41] Campos FA, Bertuzzi R, Dourado AC, Santos VG, Franchini E (2012). Energy demands in taekwondo athletes during combat simulation. Eur J Appl Physiol.

[42] Baguet A, Koppo K, Pottier A, Derave W (2010). Beta-alanine supplementation reduces acidosis but not oxygen uptake response during high-intensity cycling exercise. Eur J Appl Physiol.

[43] Lopes-Silva JP, Da Silva Santos JF, Artioli GG, Loturco I, Abbiss C, Franchini E (2018). Sodium bicarbonate ingestion increases glycolytic contribution and improves performance during simulated taekwondo combat. Eur J Sport Sci.

[44] Zoeller RF, Stout JR, O’kroy JA, Torok DJ, Mielke M (2007). Effects of 28 days of beta-alanine and creatine monohydrate supplementation on aerobic power, ventilatory and lactate thresholds, and time to exhaustion. Amino Acids.

[45] Higgins MF, Wilson S, Hill C, Price MJ, Duncan M, Tallis J (2016). Evaluating the effects of caffeine and sodium bicarbonate, ingested individually or in combination, and a taste-matched placebo on high-intensity cycling capacity in healthy males. Appl Physiol Nutr Metab.

[46] Brisola GMP, Miyagi WE, da Silva HS, Zagatto AM (2015). Sodium bicarbonate supplementation improved MAOD but is not correlated with 200- and 400-m running performances: a double-blind, crossover, and placebo-controlled study. Appl Physiol Nutr Metab.

[47] Artioli GG, Gualano B, Coelho DF, Benatti FB, Gailey AW, Lancha AH (2007). Does sodium-bicarbonate ingestion improve simulated judo performance?. Int J Sport Nutr Exerc Metab.

[48] Saunders B, Sale C, Harris RC, Sunderland C (2014). Sodium bicarbonate and high-intensity-cycling capacity: variability in responses. Int J Sports Physiol Perform.

[49] Felippe LC, Lopes-Silva JP, Bertuzzi R, McGinley C, Lima-Silva AE (2016). Separate and combined effects of caffeine and sodium-bicarbonate intake on judo performance. Int J Sports Physiol Perform.

[50] Bishop D, Edge J, Davis C, Goodman C (2004). Induced metabolic alkalosis affects muscle metabolism and repeated-sprint ability. Med Sci Sports Exerc.

[51] Gonçalves LC, Bessa A, Freitas-Dias R, Luzes R, Werneck-de-Castro JPS, Bassini A (2012). A sportomics strategy to analyse the ability of arginine to modulate both ammonia and lymphocyte levels in blood after high-intensity exercise. J Int Soc Sports Nutr.

[52] Kantanista A, Kusy K, Zarębska E, Włodarczyk M, Ciekot-Sołtysiak M, Zieliński J (2016). Blood ammonia and lactate responses to incremental exercise in highly-trained male sprinters and triathletes. Biomedical Human Kinetics.

[53] Włodarczyk M, Kusy K, Słomińska E, Krasiński Z, Zieliński J (2019). Changes in blood concentration of adenosine triphosphate metabolism biomarkers during incremental exercise in highly trained athletes of different sport specializations. J Strength Cond Res.

[54] Hsueh C-F, Wu H-J, Tsai T-S, Wu C-L, Chang C-K (2018). The effect of branched-chain amino acids, citrulline, and arginine on high-intensity interval performance in young swimmers. Nutrients.

[55] Prado ES, Neto JM, de R, Almeida, de Melo RD, de Cameron MGD. L-C. Keto analogue and amino acid supplementation affects the ammonaemia response during exercise under ketogenic conditions. Br J Nutr. 2011;105(12):1729–33. 10.1017/S000711451000557X 21324213

[56] Bassini-Cameron A, Monteiro A, Gomes A, Werneck-de-Castro JPS, Cameron L (2008). Glutamine protects against increases in blood ammonia in football players in an exercise intensity-dependent way. Br J Sports Med.

[57] Carvalho-Peixoto J, Alves RC, Cameron L-C (2007). Glutamine and carbohydrate supplements reduce ammonemia increase during endurance field exercise. Appl Physiol Nutr Metab.

[58] Holeček M, Vodeničarovová M (2018). Effects of branched-chain amino acids on muscles under hyperammonemic conditions. J Physiol Biochem.

[59] Wilkinson DJ, Smeeton NJ, Watt PW (2010). Ammonia metabolism, the brain and fatigue; revisiting the link. Prog Neurobiol.

[60] Yuan Y, Chan KM (2000). A review of the literature on the application of blood ammonia measurement in sports science. Res Q Exerc Sport.

