# Supplementary material for: The effect of multi-ingredient intra- versus extra-cellular buffering supplementation combined with branched-chain amino acids and creatine on exercise-induced ammonia blood concentration and aerobic capacity in taekwondo athletes
Source: J Int Soc Sports Nutr. 2021 Jun 14;18:48. doi: 10.1186/s12970-021-00451-3 (PMC8204562; doi:10.1186/s12970-021-00451-3)
Supplement: Supplementary file 1 — Additional file 1: Supplementary Table S1. The number, duration, and average intensity (percentage of maximum heart rate) of training sessions and tournaments in tested taekwondo athletes before, during the supplementation and washout periods. [file 12970_2021_451_MOESM1_ESM.pdf]

Supporting files

[Table S1] The number, duration, and average intensity (percentage of maximum heart rate) of training sessions and tournaments in tested taekwondo athletes before, during the supplementation and washout periods.

	**Initial period*** 8 weeks 5 Dec 2015 – 29 Nov 2015		**1st Treatment** 8 weeks 30 Nov 2015 – 15 Jan 2016			**Washout** 8 weeks 16 Jan 2016 – 17 Apr 2016			**2nd Treatment** 8 weeks 18 Apr 2016 – 12 Jun 2016		
	No.	Hours	No.	Hours	Intensity	No.	Hours	Intensity	No.	Hours	Intensity
Training sessions											
- total	44	88	49	98	–	56	112	–	41	82	–
- taekwondo-specific circuits	11	22	16	32	90±2	19	38	91±2	8	16	90±2
- speed & power	–	–	–	–	–	6	12	–	8	16	–
- technique & tactics	9	18	17	34	83±3	16	32	85±3	17	34	88±2
- strength & conditioning	12	24	–	–	–	–	–	–	–	–	–
- individual (strength or technique or endurance)	12	24	16	24	–	15	30	–	8	12	–
Tournaments	4	–	4	–	93±1	5	–	94±1	6	–	93±1

* workout intensity was not recorded in this period
